# Girls with Premature Thelarche Younger than 3 Years of Age May Have Stimulated Luteinizing Hormone Greater than 10 IU/L

**DOI:** 10.4274/jcrpe.galenos.2020.2019.0202

**Published:** 2020-11-25

**Authors:** Gülcan Seymen Karabulut, Müge Atar, Filiz Mine Çizmecioğlu Jones, Şükrü Hatun

**Affiliations:** 1 Ümraniye Training and Research Hospital, Clinic of Pediatric Endocrinology, İstanbul, Turkey; 2 Süleyman Demirel University Faculty of Medicine, Department of Pediatric Endocrinology, Isparta, Turkey; 3 Kocaeli University Faculty of Medicine, Department of Pediatric Endocrinology, Kocaeli, Turkey; 4 Koç University Faculty of Medicine, Department of Pediatric Endocrinology, İstanbul, Turkey

**Keywords:** GnRH stimulation test, central precocious puberty, young girls

## Abstract

**Objective:**

Premature thelarche (PT) is defined as isolated breast development in girls before eight years of age. Gonadotropin-releasing hormone (GnRH) stimulation test is sometimes used to distinguish between PT and central precocious puberty (CPP), although the interpretation of the test at early ages is challenging. The objective of this study was to determine the follicle stimulating hormone (FSH) and luteinizing hormone (LH) responses to GnRH stimulation in girls with PT below 3 years of age.

**Methods:**

A standardized GnRH stimulation test, bone age and pelvic ultrasound were evaluated and those without pubertal progression after a minimum of one-year follow up were included in the study.

**Results:**

On GnRH stimulation test, the median (range) baseline LH was 0.29 (0.10-0.74) IU/L, baseline FSH was 4.96 (3.18-7.05) mIU/mL, and the peak median LH was 5.75 (3.31-8.58) IU/L with the peak mean±standard deviation FSH was 40.38±20.37 mIU/mL. Among the patients, 33.3% (n=10) had baseline LH >0.3 IU/L, 67% (n=20) had peak LH >5 IU/l and 16.6% (n=5) >10 IU/L. The mean peak LH/FSH ratio was 0.17±0.09 and was ≤0.43 in all participants.

**Conclusion:**

Although consensus statements usually define baseline LH >0.3-0.5 IU/L, peak LH >5 IU/L, and LH/FSH ratios >0.66-1.0 as diagnostic cut-offs for CPP, in children below 3 years of age, the baseline and peak LH values may be similar to pubertal values, possibly due to mini-puberty. A dominant FSH response on GnRH stimulation test is more valuable than the peak LH response in the diagnosis of PT.

**What is already known on this topic?**

A gonadotropin-releasing hormone (GnRH) stimulation test is the gold standard for the diagnosis of central precocious puberty and a peak stimulated luteinizing hormone (LH) level of 5 mIU/L is considered pubertal by many endocrinologists.

**What this study adds?**

GnRH stimulation test reference values differ in young girls with premature thelarche (PT) compared to older girls. In all girls with PT under three years of age, peak LH/follicle stimulating hormone ratio was ≤0.43, regardless of peak LH levels which were usually >5 IU/L in two thirds and were even >10 IU/L in some girls.

## Introduction

Precocious puberty (PP) has been an increasing concern in recent years due to both an increase in the number of related outpatient visits and the existing challenge of determining the patients who require treatment. Most girls with signs of puberty in the first three years of life are diagnosed with premature thelarche (PT), which is a benign and non-progressive condition. While early skeletal maturation, increase in height growth rate and decrease in adult height are seen in central PP (CPP), these findings are not seen in PT. How often girls initially diagnosed with PT progress to CPP and how much monitoring is required is contentious. Certain clinicians perform gonadotropin-releasing hormone (GnRH) stimulation tests based on the clinical findings and/or randomly requested baseline hormonal tests in these patients (1,2). The consensus statements on PP recommend to use the same threshold values in the interpretation of the GnRH stimulation tests in all children below eight years of age. These thresholds values are a stimulated luteinizing hormone (LH) >5 IU/L or a LH/follicle stimulating hormone (FSH) ratio above 0.66 or 1.0; these results support a diagnosis of CPP (3,4,5). However, patients in the younger age group present challenges in the interpretation of diagnostic tests, due to the impact of the activation of the hypothalamic-pituitary-gonadal axis in the first months of life, termed ‘mini-puberty’ (6). Although a limited number of studies report that higher stimulated LH responses may be observed following GnRH stimulation tests conducted in children below three years old (7), this information is not highlighted in consensus statements (1,8).

In the present study, GnRH stimulation test results of children diagnosed with PT before the age of three years were evaluated and the validity of the criteria used in pediatric age groups to distinguish between pubertal and prepubertal responses was investigated.

## Methods

Thirty girls with PT under the age of three years, admitted to the Pediatric Endocrinology Department outpatient clinic of Kocaeli University, between January 2010 and June 2016, were prospectively evaluated. All parents received oral and written information before being asked for consent. The study was approved by the Local Ethics Committee of Kocaeli University Institutional Review Board (KÜ GÖKAEK 2016/70).

PT was diagnosed based upon the following criteria: isolated breast development without other signs of puberty; and bone age within ±2 standard deviation (SD) of mean for chronological age. The diagnosis of PT was confirmed by a lack of pubertal progression over at least one-year follow-up. Signs suggestive of pubertal progression were growth acceleration with a height velocity >1 SD score (SDS) and/or progression to Tanner breast stage >3 and/or bone age acceleration.

During the initial assessment of the girls presented with isolated breast development, a detailed patient history was taken and a physical examination was performed. The length of the children aged <2 years of age was measured in the supine position on the head-foot board (Seca Limited, Hammer Steindamm, Hamburg, Germany), while the height of children >2 years of age was measured in a standing position using a Harpenden Stadiometer (Holtain Limited, Crymych, Dyfed, UK). The SDS for height was calculated based on Turkish Child growth standards (9). Height velocity SDS was calculated using Tanner’s growth charts (10). Puberty stage was assessed by physical examination according to Tanner’s criteria for breast development in females (11). The left hand and wrist X-rays for bone aging, pelvic ultrasonography (USG), and GnRH stimulation tests were performed at the time of the diagnosis. Bone age was assessed using the Greulich & Pyle method by the same pediatric endocrinologist and repeated every 6 to 12 months (12). Bone age acceleration was defined as Δ bone age/Δ calendar age >1. Longitudinal diameter of the uterus >34 mm at pelvic USG was defined resulting from estrogen exposure (13).

### GnRH Stimulation Test Procedure

An intravenous (IV) cannula was inserted into the antecubital region for blood sampling and GnRH analogue injection. After the baseline blood was drawn for LH and FSH measurement, gonadorelin acetate (LH-RH ferring ampul, 0.1 mg/mL, Ferring İlaç San. ve Tic. Ltd., İstanbul, Turkey) 0.1 mg/m^2^ body surface area (max 0.1 mg) was injected as an IV bolus and a second blood sample was obtained at the 40^th^ minute (14). LH and FSH were measured using an immuno-chemiluminescense assay using an Immulite 1000 apparatus and commercial kits (Diagnostic Products Corp.-Medlab, Los Angeles, CA) (15). For LH, the intra- and inter-assay coefficients of variation (CVs) were 4.8% and 10.7%, respectively. For FSH, the intra- and inter-assay CVs were 3.4% and 5.4%, respectively. The minimum detectable concentration was 0.1 IU/L for both FSH and LH.

### Statistical Analysis

Statistical evaluation was performed using the SPSS, version 20.0 (IBM Inc., Chicago, IL, USA). The normality of the distribution was assessed with the Kolmogorov-Smirnov Test. Numeric variables were expressed as mean +/- SD and median plus 25^th^ to 75^th^ percentile (interquartile range) and frequency (percentage). Independent t-tests were used when comparing continuous variables that were normally distributed. Continuous variables that were not normally distributed were assessed using the Mann-Whitney U test. Categorical variables were presented as numbers and percentages. Differences between categorical variables, such as Tanner stage, were assessed using a chi-square test. The relationship between the variables within the normal distribution was evaluated using Pearson’s correlation analysis, while the relationship between the variables outside the normal distribution was analysed through Spearman’s correlation analysis. Statistical significance was assumed with a value of p<0.05.

## Results

**Figure 1:**
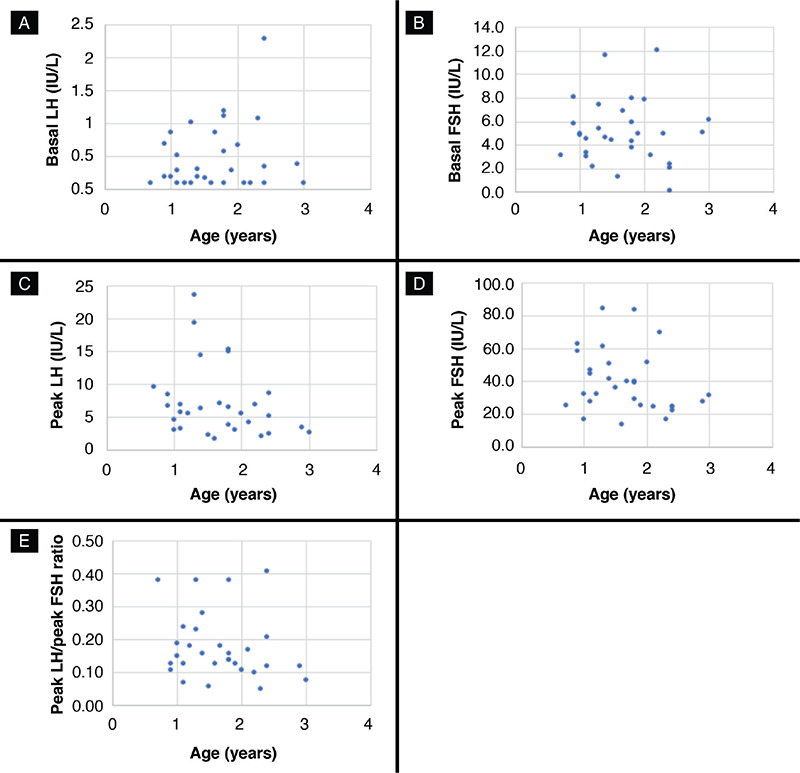
A) The distribution of the baseline luteinizing hormone (LH) levels according to age. B) The distribution of the peak LH responses according to age. C) The distribution of the baseline follicle-stimulating hormone (FSH) levels according to age. D) The distribution of the peak FSH responses according to age. E) The distribution of the peak LH/ peak FSH ratio according to age

Thirty girls aged under three years with PT, in whom the diagnosis was confirmed by lack of pubertal progression in at least one-year of follow-up, were enrolled in the study. Clinical characteristics of patients are shown in Table 1. The bone age was within mean ±2 SD of chronologic age range in all the patients. The pelvic USG results showed that the uterine sizes and ovarian volumes were consistent with the ages of the patients and no pathologies were observed. The results of the GnRH stimulation tests are presented in Table 2. The distribution of the baseline LH and FSH, peak LH and FSH, and the peak LH/FSH ratios according to age are shown in Figure 1. The baseline LH value was >0.3 IU/L in 10/30 patients (33.3%). In 67% of the patients (n=20), the peak LH value was >5 IU/L, while it was >10 IU/L in 16.6% (n=5). In all patients, the peak LH/FSH ratio was ≤0.43.

No significant relationship was observed between the baseline LH and the peak LH (r=0.054, p=0.776). However, there was a positive correlation between stimulated FSH and stimulated LH (r=0.647, p<0.001).

There were no significant differences between subjects with basal LH values >0.3 IU/L and <0.3 IU/L in terms of Tanner breast stage, bone age, basal FSH, estradiol concentration, peak LH and peak FSH values (Table 3). Similarly, there were no significant differences in Tanner stage, bone age, basal LH, basal FSH, estradiol concentration and peak FSH values between the groups with peak LH value >5 IU/L and peak LH value <5 IU/L (Table 4).

## Discussion

Signs of puberty, especially breast development, in girls up to 3 years of age are a source of concern both for the parents and physicians. At such an early age, isolated PT is the most likely diagnosis and often all that is needed is observation. On the other hand, CPP, although rare, is also observed in this age group or cases diagnosed initially as isolated PT may progress to CPP (1,16). Clinically, breast development in patients with isolated PT is unaccompanied by areola and nipple maturation, breast development shows fluctuations, and no growth spurt is observed (16). However, in a study from Israel comprising the follow up of 139 patients with PT for a decade, CPP was reported in 13%, regardless of the age of the diagnosis and the clinical progression (17). In this study, cases progressing to CPP were significantly less frequent in patients under the age of two compared with girls over 2 years of age (3.8% vs. 52.6%). Other studies from Italy and Denmark also showed 14% of girls with an initial diagnosis of PT progressed to CPP on follow-up (18,19). It is therefore warranted to perform further studies, including GnRH stimulation tests, in certain cases. However, the lack of established reference values for GnRH stimulation test responses in girls below three years taking into account the effect of mini-puberty has led to some clinical confusion. In a study from Italy including 450 patients, progression to CPP was observed in only 2% of the patients diagnosed with PT below the age of two and the baseline hormone levels, including the GnRH test, were found to be unhelpful in predicting progression (2). In this study, 97 patients were evaluated through endocrine tests and imaging methods in addition to a 3-month clinical follow up and 85 patients were diagnosed with PT, nine patients were diagnosed with CPP and three were attributed the diagnosis of peripheral PP. Among the patients with a final diagnosis of PT, 36.4% had peak LH levels >5 IU/L (100% among the patients with CPP). On the other hand, baseline LH values >0.2 IU/L were observed in only 1.17% of the patients with PT. All girls with isolated thelarche had a FSH predominant response with peak LH/FSH ratio <1, while girls with complete sexual development showed a ratio >1. A study from Taiwan also reported similar results (7). In this study, 36 patients with the final diagnosis of isolated PT were classified into two groups; patients under the age of 4 (group A) and over the age of 4 (group B). Their GnRH stimulation test results were compared. In group A, the peak mean LH was 13.0±6.06 IU/L, while it was 8.5±4.10 in group B and the peak LH response was significantly higher in group A compared to group B (p<0.05). In addition, the peak mean FSH in group A was 120.5±45.87, while it was 48.7±24.05 IU/L in group B and the peak FSH response was significantly higher in group A compared to group B (p<0.001). The peak LH/FSH ratio was <1 in all the patients.

Vestergaard et al (20) investigated the physiological LH, FSH and LH/FSH response to GnRH stimulation test in healthy girls below six years of age with no signs of PP. The study showed an age-dependent response to the GnRH test with larger LH and FSH responses in girls aged from 10 months to 3 years compared to girls aged from 3-6 years (the 30-min LH response 5.2±4.0 and 2.9±2.5 IU/L, the 30-min FSH response 23.3±16.2 and 14.5±10.3 IU/L). The peak LH/FSH ratio was 0.23±0.19 (range 0.06-0.43).

In the present study, the ratio of both the baseline LH values >0.3 IU/L (33%) and the peak LH values >5 IU/L (67%) were high. Also, 16.6% of the patients (n=5) had values of peak LH >10 IU/L. These latter patients can easily be misdiagnosed with CPP with a traditional approach. Indeed, GnRH analogue therapy had been suggested for some girls referred to our clinic based on these results, However, all patients had peak LH/FSH ratio values ≤0.43.

The main focus of our study was to analyse GnRH stimulation test results among patients with a confirmed diagnosis of isolated PT. The results showed that high gonadotropin response, even when peak LH value was >10 IU/L, was not associated with progression to true PP in our cohort as all girls had confirmed isolated PT. Thus the treatment decision should not be based solely upon these criteria. The peak LH/FSH ratio appears to be a useful parameter for defining pubertal activation. Indeed, both our study results and other studies have demonstrated that after mini-puberty, although the baseline LH and FSH values are undetectable, a GnRH test may induce a “pubertal gonadotropin response” in girls under the age of three years (2,6). Although the association of this response with the etiology of PT is yet to be elucidated, it may be a crucial diagnostic factor.

### Study Limitations

As the study was conducted as an observation of young girls with PT, the number of participants was restricted. Further studies with larger sample sizes may yield more definitive results. Also, it was not possible to compare PT patients with CPP patients or healthy girls with no signs of pubertal progression in the age group of interest because of either insufficient numbers or ethical issues.

## Conclusion

In conclusion, a peak LH value, even if >10 IU/L, is inadequate to make an accurate therapy decision in patients with PT below three years of age. Elevated LH responses to GnRH stimulation test are common, but not related to PP. The diagnosis of CPP solely based on the response to a GnRH test is often misleading in the first three years of life, leading to overestimation of CPP. In our opinion the peak LH/FSH ratio is more valuable for distinguishing between a pubertal and a prepubertal response. As highlighted in recent years, there is still uncertainty regarding the diagnosis and treatment of early puberty, and the evaluation of the biochemical results with the clinical findings and the age of the patient is one of the most important points to avoid unnecessary treatment (21,22).

## Figures and Tables

**Table 1:**
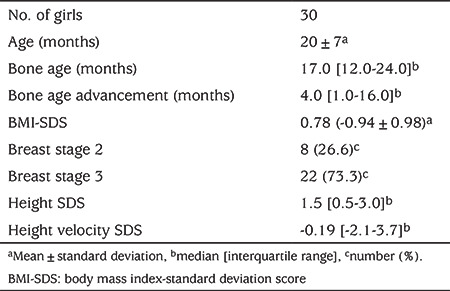
Clinical characteristics of girls with premature thelarche

**Table 2:**
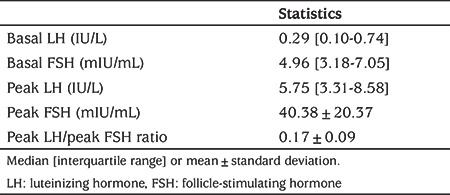
Hormonal results following gonadotropinreleasing hormone stimulation test in girls with premature thelarche

**Table 3:**
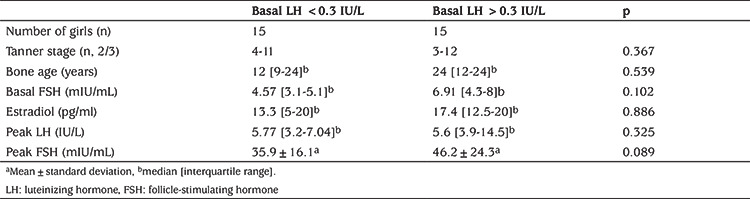
The comparison of girls with basal luteinizing hormone value <0.3 IU/L and >0.3 IU/L

**Table 4:**
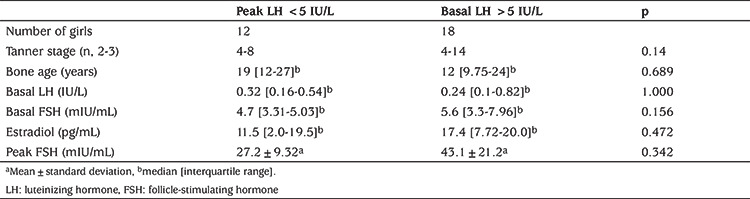
The comparison of girls with peak luteinizing hormone value <5 IU/L and >5 IU/L
